# Access to the COVID-19 Vaccine

**DOI:** 10.3390/ijerph191711054

**Published:** 2022-09-03

**Authors:** Dimitris Zavras

**Affiliations:** Laboratory for Health Technology Assessment, Department of Public Health Policy, School of Public Health, University of West Attica, 11521 Athens, Greece; dzavras@uniwa.gr

As of 31 August 2022, 599,825,400 confirmed coronavirus disease 2019 (COVID-19) cases and 6,469,458 deaths have been reported globally [1]. The pandemic has tested humanity for more than two years. What characterizes this time period is the high death toll, the suffering [2], and the high levels of uncertainty in every aspect of the crisis. In the early phase of the pandemic, the time needed to develop and deploy vaccines was one of the sources of such uncertainty [3].

Due to the importance of COVID-19 vaccination in saving lives and in contributing to global economic recovery, this Editorial attempts to study the access to the COVID-19 vaccine.

The world’s first vaccination was made available in December 2020 [4]. Reducing the spread of the disease, protecting segments of the population that are not yet vaccinated as well as those for whom vaccines have limited efficacy, and reducing severe cases that burden healthcare systems are among the benefits of vaccination against COVID-19 [5]. In addition, economic recovery will be faster in countries with higher vaccination rates [6].

However, although healthcare access is a right, there are still more than two and a half billion people around the world who are unvaccinated [4], and access to vaccines in low- and middle-income countries (LMICs) is far lower than it is in high-income countries (HICs). Consequently, rates of COVID-19 infections and deaths are higher in LMICs [7]. 

It is evident that access to the COVID-19 vaccine is inequitable; such global inequity is due to the concentration of vaccine development and production in high-income countries, dose-hoarding by those countries, high vaccine prices, and challenges in deploying vaccines in resource-poor settings [8]. Beyond these reasons, the existence of barriers in terms of access to healthcare may undermine the achievement of health equity [9]. According to Mooney [10], “if we are taking access to mean opportunity to use or freedom to use, it is immediately apparent that it is the potential users’ perspectives on what constitutes a barrier and its height that must be used in assessing differential access”, while “freedom to use describes the social possibility and the individual ability to give direction to one’s will to use health services” [11].

Given that “equal access” is defined in parallel with “equal need”, a question is posed: how should need be approached? The answer is that need can be considered as an expression of the risk of contracting COVID-19 [12]; people aged 60 years and over, those living in long-term care facilities, and people with underlying health conditions such as hypertension, diabetes, cardiovascular disease, etc., are considered high-risk groups for COVID-19 [13].

On the other hand, access should be approached through its dimensions and through the barriers that individuals face.

Adopting Penchansky’s and Thomas’s [14] framework for the dimensions of access, i.e., the five As, namely, availability, affordability, accessibility, accommodation, and acceptability, the global picture regarding the COVID-19 vaccine is as follows: (a) the availability of COVID-19 vaccines differs significantly around the world [15], and specifically, in LMICs, availability is far lower than in wealthier countries [16]; (b) in several countries, COVID-19 vaccines are provided free of charge [17], and thus, their affordability may not be an issue in monetary terms, something that is not true in some less developed nations [18]; (c) for several communities around the world, accessibility is an issue [19,20]; (d) telehealth has been adopted on a large scale for vaccination registration and for relevant medical interventions, but knowing how to further improve vaccination accommodation through telehealth while bridging the digital divide is another matter that requires immediate investigation and implementation [21]; and (e) while developing countries mainly face affordability—and accessibility-related issues regarding vaccines, developed countries face acceptability issues [22,23].

With regard to the acceptance or refusal of the COVID-19 vaccine, vaccine hesitancy is a major obstacle to high vaccination rates [24]. According to Lin et al. [25], the most common reasons for vaccination hesitation or refusal are a fear of side effects; safety; and effectiveness, while the belief that the vaccines are unnecessary; inadequate information; the unknown/short duration of vaccines; and a general anti-vaccine stance are associated with lower acceptance. In addition, vaccine acceptance on a global scale is influenced by the speed of vaccine development [26].

A fear of side effects, safety or effectiveness concerns, and beliefs related to the COVID-19 vaccine are demand-side barriers; concerns related to vaccination timing, vaccination location, and the financial cost of getting vaccinated are also demand-side barriers. Vaccine delivery and administration barriers, such as inaccessible places and people, are supply side barriers [27]. 

Significant predictors of COVID-19 vaccine uptake intentions are education, the existence of insurance coverage, scoring high on subjective norms, a positive attitude toward the vaccine, high perceived susceptibility to COVID-19, high perceived benefits of the vaccine, and scoring low on barriers to the vaccine [28].

Holloman [29] defined access to healthcare as the freedom from barriers to healthcare, while barriers are defined as anything that constrains, deters, delays, denies, dissuades, discourages, handicaps, or prevents the acquisition or utilization of those services that are ultimately provided by society to its members individually and collectively for the maintenance, preservation, and improvement of health.

In the case of COVID-19 vaccination, the “freedom to receive a vaccination” has been undermined to a large extent by exposure to misinformation [30]. However, we should note that a belief in misinformation may be related to prevailing generalized uncertainty. COVID-19-related uncertainty arose from various aspects of the COVID-19 crisis, i.e., health-related, economic, and social. Uncertainties related to the nature of the virus, alternative treatment options, clinical outcomes, and prevention methods [31] all concern the health dimension of the crisis. Uncertainties related to impacts on personal finances and the effectiveness and impacts of the restriction measures [32] concern the economic dimension of the crisis.

As mentioned above, barriers play a decisive role in access to healthcare, and in the actual use of services [33]. In addition, since awareness, i.e., communication and information, may be considered as the sixth dimension of access [34], providing up-to-date information would be an effective strategy against barriers such as fear or concerns related to the safety and effectiveness of the vaccine [35]. Since vaccination rates have increased in numerous communities as populations have begun to see their friends, colleagues, and neighbors get vaccinated without adverse events [36], relative information could effectively motivate individuals to get the vaccine.

COVID-19 vaccination concerns all of us, and efforts to minimize barriers to receiving it should continue. Thus, understanding such barriers and the best way to influence individuals is crucial to achieve high vaccination rates. The affordability and accessibility issues facing developing countries and the acceptability issues facing developed countries must be eliminated. “Freedom to receive a vaccination” should be translated to freedom to fight the enemy that threatens one’s life, health, and well-being, and the global economy. 
